# Editorial: Biofilm formation and quorum sensing of foodborne microorganism

**DOI:** 10.3389/fmicb.2022.1107603

**Published:** 2022-12-13

**Authors:** Lili Li, Tao Yu, Lei Yuan, Agapi I. Doulgeraki, Ramona Iseppi

**Affiliations:** ^1^Institute of Food Safety and Nutrition, Jinan University, Guangzhou, China; ^2^School of Life Sciences and Basic Medicine, Xinxiang University, Xinxiang, China; ^3^School of Food Science and Technology, Yangzhou University, Yangzhou, China; ^4^Institute of Technology of Agricultural Products, Hellenic Agricultural Organization Lycovrissi, Lycovrissi, Greece; ^5^Department of Life Sciences, University of Modena and Reggio Emilia, Modena, Italy

**Keywords:** biofilm, quorum sensing, food safety, control, pathogen

Biofilms are a self-protection growth pattern of microorganisms and are commonly defined as communities of microbial cells enclosed in hydrated extracellular polymeric substances and adherent to surfaces (Sauer et al., 2007). Biofilm cells are more resistant to cleaning and disinfection processes in the food industry (Yuan et al., 2021). Therefore, biofilms represent an important source of contamination of raw materials and processed products, posing a serious threat to food safety.

Biofilm formation is a complex process influenced by many factors. Quorum sensing (QS) is a cell-to-cell communication process that allows microorganisms to behave coordinately in response to environmental changes by producing, secreting, and detecting signal molecules (Bassler, 1999; Subramani and Jayaprakashvel, 2019). Previous studies have confirmed that QS plays a significant role in biofilm formation (Zhou et al., 2020) and is vital for food spoilage and food-related pathogenesis (Machado et al., 2020). Understanding of mechanisms behind QS and biofilm formation and exploring control strategies are important to enhance food safety.

In this context, this Research Topic aims to collect recent studies on the following themes:(1) The mechanisms underlying biofilm formation of food microbiology; (2) The role of QS in biofilm formation, food spoilage, and food-related pathogenesis; (3) The novel strategies for biofilm control in food microbiology; (4) Identification of QS inhibitors in food microbiology; (5) QS interfering mechanisms in food microbiology. This Research Topic comprises 8 original research articles from Israel, China, Mexico, Ireland, Iran, and Finland, contributed by 58 authors. Most of contributions focused on the mechanisms underlying biofilm formation, one developed a novel natural antimicrobial substance for biofilm control and one overviewed the literature relating to QS from the perspective of the interactions between the food and human gut microbiome.

The mechanisms underlying biofilm formation of a large number of important foodborne pathogens are still largely unknown. Li et al. investigated the mechanism of biofilm formation in emetic *Bacillus cereus* strains by random mutagenesis and confirmed the dual role of the flagellar hook gene *flgE* in the biofilm formation and cereulide production in emetic *B. cereus*. Cheng et al. determined the role of SdiA in biofilm formation and pathogenicity in *Cronobacter sakazakii* by gene editing technology. They revealed that SdiA enhanced the drug resistance of *C. sakazakii* and suppressed biofilm formation, as well as motility and adhesion. Moreover, Zhang et al. investigated the regulatory function of RpoS on spoilage activity and adhesion ability in *Shewanella baltica* and demonstrated that RpoS is a primary regulator involved in flagellar assembly mediated biofilm formation and cold adaptation-related spoilage activity of *S. baltica*. The results of these studies provide significant insights into the mechanisms underlying biofilm formation and control of bacterial infection.

Biofilm formation is influenced by many factors. Suissa et al. systematically compared five *Lactobacillaceae* strains for the effects of different carbohydrates on their free-living and biofilm lifestyles and indicated that the formation of biofilms and aggregation capacity were responsive to the carbohydrate provided. Avila-Novoa et al. demonstrated that the proportion of components that make up the extracellular matrix are associated with factors such as culture media (less nutrient-rich laboratory medium and supplements of medium) and genetic characteristics of the *Staphylococcus aureus* isolates. Moreover, Zarei et al. analyzed interaction between different foodborne pathogens in dual-species biofilms and illustrated that *Pseudomonas* biofilms may attract and/or shelter other spoilage or pathogenic bacteria, which is of great concern for the dairy industry.

Regarding the role of QS in biofilm formation, Falà et al. summarized and critically discussed the literature, providing a general overview of the current understanding of the prevalence and influence of QS on biofilms, interactions with components of food matrices and host-associated factors in the human gut.

From all the above it seems that novel strategies for biofilm control in food microbiology are urgently needed. To this point, Liu and Wang explored the effects of protocatechuic aldehyde on the biofilm formation and adhesion capabilities of *Vibrio parahaemolyticus*. The results of this study demonstrated that protocatechuic aldehyde can be used to control *V. parahaemolyticus* biofilm to ensure food safety.

The above studies have expanded our understanding on this topic; however, relevant studies about (1) the mechanisms underlying biofilm formation of different bacterial species, (2) the role of QS in biofilm formation and (3) novel strategies for QS interfering and biofilm control still needed for a better understanding of bacterial biofilms.

## Author contributions

All authors listed have made a substantial, direct, and intellectual contribution to the work and approved it for publication.
