# Corrigendum: Editorial: Biofilm formation and quorum sensing of foodborne microorganism

**DOI:** 10.3389/fmicb.2023.1144058

**Published:** 2023-02-09

**Authors:** Lili Li, Tao Yu, Lei Yuan, Agapi I. Doulgeraki, Ramona Iseppi

**Affiliations:** ^1^Institute of Food Safety and Nutrition, Jinan University, Guangzhou, China; ^2^School of Life Sciences and Basic Medicine, Xinxiang University, Xinxiang, China; ^3^School of Food Science and Technology, Yangzhou University, Yangzhou, China; ^4^Institute of Technology of Agricultural Products, Hellenic Agricultural Organization Lycovrissi, Lycovrissi, Greece; ^5^Department of Life Sciences, University of Modena and Reggio Emilia, Modena, Italy

**Keywords:** biofilm, quorum sensing, food safety, control, pathogen

In the published article, there was an error in affiliation 5.

Instead of “Institute of Technology of Agricultural Products, Hellenic Agricultural Organization – DIMITRA, Lycovrissi, Attica, Greece,” it should be “Department of Life Sciences, University of Modena and Reggio Emilia, Modena, Italy.”

The authors apologize for this error and state that this does not change the scientific conclusions of the article in any way. The original article has been updated.

